# Editorial: Dendritic spines: from shape to function^†^

**DOI:** 10.3389/fnana.2015.00101

**Published:** 2015-07-28

**Authors:** Nicolas Heck, Ruth Benavides-Piccione

**Affiliations:** ^1^Sorbonne Universités, UPMC Paris 06, Institut de Biologie Paris Seine, Neuroscience Paris SeineParis, France; ^2^Instituto Cajal (CSIC)Madrid, Spain; ^3^Laboratorio Cajal de Circuitos Corticales (CTB), Universidad Politécnica de MadridPozuelo de Alarcón, Spain

**Keywords:** dendrites, pyramidal cell, cerebral cortex, synapses, synaptic integration

In 1888, Cajal discovered the existence of thin protrusions emerging from the surface of certain neurons. He proposed that these protrusions, which he called dendritic spines, could correspond to points of contact between neurons and beautifully illustrated them with elegant drawings. However, it was not until 1959, with the introduction of electron microscopy, that Gray made the definitive observation which confirmed that dendritic spines are indeed postsynaptic structures that establish synaptic contacts with axon terminals. Since then, spines have received much attention, as they are the major targets of excitatory synapses in the brain. Their density and morphology appear to be indicative of the cellular processes involved in neural plasticity which correlate with cognitive functions such as learning and memory, and are symptomatic in several neuropathologies such as mental retardation and neurodegenerative diseases. Thus, they are believed to be functional integrative units, with a morphology that is closely related to their function, that play an integral role in the activity of spiny cells, yet their exact function still remains unclear. This research topic “Dendritic spines: from shape to function” contains 20 articles that aim to capture the current state of this research field, bringing together some of the latest relevant findings regarding dendritic spine structure and function. It combines 11 reviews, 3 minireviews, 2 original research articles, 3 method articles, and 1 perspective article. Out of the 79 authors that participated in this volume, we would like to dedicate this research topic to Dr. Dominique Muller, a magnificent scientist that contributed to this research topic and regrettably passed away on April 29th 2015 in an accident (Lüscher et al., 2015). He was strongly involved in the study of the molecular mechanisms of synaptic network remodeling as well as in synaptogenesis research, providing insight into how synaptic plasticity contributes to brain repair. Indeed, in the present research topic, he and his co-authors review some of these recent advances and discuss the hypothesis that alterations of structural plasticity could represent a common mechanism contributing to the cognitive and functional defects observed in diseases such as intellectual disability, autism spectrum disorders and schizophrenia (Bernardinelli et al., 2014).

The additional articles included in this volume are briefly summarized below:

A historical review by DeFelipe focuses on the discovery of dendritic spines by Cajal, which was possible thanks to the application of the Golgi technique to the study of the nervous system. He highlights how this discovery represents an interesting chapter in the history of neuroscience as it shows us that progress in the study of the structure of the nervous system is based not only on the emergence of new techniques, but also on our ability to exploit the methods already available to correctly interpret microscopic images (DeFelipe, 2015).

Araya's review highlights relevant findings, challenges and hypotheses regarding spine function, with an emphasis on the electrical properties of spines and how these affect the storage and integration of excitatory synaptic inputs in pyramidal neurons. He proposes that dendritic spines exist due to their ability to undergo activity-dependent structural and molecular changes that are able to modify synaptic strength, and hence alter the gain of the linearly integrated sub-threshold depolarizations in pyramidal neuron dendrites before the generation of a dendritic spike (Araya, 2014).

Hoogenraad and Kapitein groups review dendritic spine morphology and compartmentalization. The authors review recent advances in tools development that facilitate studying the role of the spine neck in compartmentalization. In particular, spatial restriction of signaling, constraints on molecules and electrical signal diffusion are discussed, as are constraints on receptor mobility. The review also covers the methodological challenges that live-cell imaging of spines implies (Adrian et al., 2014).

Stephen Wong laboratory discusses the opportunities for analysis of neuronal spine anatomy and function provided by new imaging technologies and the high-throughput application of older technologies, while evaluating the strengths and weaknesses of currently available computational analytical tools (Mancuso et al., 2014).

Frotscher et al. unravel the ultrastructure of the spine. The history of electron microscopy studies on spines is given, and advantages and pitfalls of the method are reported. This article gives insight into the high-pressure freezing technique that avoids artifacts from chemical fixation. The technique is illustrated with original data on subtle fine structural changes in spine shape associated with chemically induced long-term potentiation (Frotscher et al., 2014).

Segal and Korkotian review the endoplasmic reticulum calcium stores in dendritic spines. Several key issues are addressed, including the role of calcium stores in synaptic plasticity, their role during development, in stress and in neurodegenerative diseases. This review gathers together the evidence for a crucial role of calcium stores in synaptic plasticity and neuronal survival (Segal and Korkotian, 2014).

Dotti et al. review the current knowledge on lipid dynamics at dendritic spines. The functional implication of lipid metabolic enzymes on synapse function is discussed, revealing critical roles in synapse physiology and pathology. The current knowledge on the regulation of glutamate receptors by lipid associated signaling is also reviewed (Dotti et al., 2014).

Koleske's group reviews the importance of the surrounding extracellular matrix for the regulation of spine formation and synapse maintenance and plasticity, with detailed descriptions of the function of several matrix proteins. Their review also covers the role of proteases specific to matrix proteins (Levy et al., 2014).

The regulation of spine structural plasticity by matrix metalloproteinase-9 is also the subject of the review by Wlodarczyk's team, who propose the term tetrapartite synapse to emphasize the relevance of the matrix as an integral component of synapse regulation. Additionally, the authors examine the critical role of matrix proteases in spine alterations observed in epilepsy (Stawarski et al., 2014).

Elston and Fujita review recent findings related to postnatal spinogenesis; dendritic and axon growth; pruning; and electrophysiology in neocortical pyramidal cells in the developing primate brain. They correlate anatomical findings with electrophysiological properties of cells in different cortical areas. These authors suggest that the anatomical and electrophysiological profiles of pyramidal cells vary among cortical areas at birth, and continue to diverge into adulthood (Elston and Fujita, 2014).

Zuo's group summarizes in a minireview *in vivo* studies which assess spine dynamics in correlation with brain development; sensory experience; learning and memory; and pathologies. The question of whether spines emerge at random places on the dendrite or in clusters is discussed, and future directions for the field are proposed (Chen et al., 2014).

Spiga et al. report on the connectivity changes and spine morphology modifications that accompany drug addiction. This minireview of the literature emphasizes how different substances which all lead to addiction have very different effects on spines and how a same substance can differentially modify spines depending on the administration protocol and timing of the observation (Spiga et al., 2014).

Pozzo-Miller's group focus on the link between Rett syndrome, which arises from loss of function mutations of the transcription factor MECP2, and spine alterations. In this minireview the molecular exploration of the basis of spine phenotype allows the authors to develop a therapeutic approach centered on the neurotrophic factor BDNF (Xu et al., 2014).

Takasaki and Sabatini describe in an original article the application of 2-photon microscopy combined with stimulated emission depletion (STED-2P) to the biophysical study of the relationship between synaptic signals and spine morphology, demonstrating the utility of combining STED-2P with modern optical and electrophysiological techniques. These authors identify and evaluate morphological determinants of fluorescence recovery time within the context of a simple compartmental model describing diffusive transfer between spine and dendrite. They also investigate correlations between the neck geometry and the amplitude of synaptic potentials and calcium transients evoked by 2-photon glutamate uncaging (Takasaki and Sabatini, 2014).

Majewska's group investigate in an original article whether a low dose exposure of Bisphenol-A (a monomer used in the production of many common household objects, BPA) during a developmental phase when brain connectivity is being organized can cause long-term deleterious effects on brain function and plasticity. The authors use immunohistochemistry to examine histological markers known to impact cortical maturity and developmental plasticity. They quantify cortical dendritic spine density, morphology, and dynamics suggesting that exposure to very low levels of BPA during a critical period of brain development can have profound consequences for the normal wiring of sensory circuits and their plasticity later in life (Kelly et al., 2014).

The method articles propose state-of-the-art technical improvements which bring spine research to the next level. Cheng et al. specifically describe a method to fluorescently label and visualize dissociated hippocampal neurons using the fluorescent marker DiI (a carbocyanine membrane dye that exhibits enhanced fluorescence upon insertion of its lipophilic hydrocarbon chains into the lipid membrane of cells) and high-resolution confocal microscopic imaging. This method labels neuronal and synaptic morphology to permit quantitative analysis of dendritic spines (Cheng et al., 2014).

The method article by Holtmaat's team provides further *in vivo* imaging of spines with a method that allows the observation of specific protein markers of the synapse. *In vivo* single cell electroporation is used for the neuron to express markers such as PSD95, and the marked synapse is observed *in vivo* over long periods of time. Importantly, the markers show differential kinetics on the same dendrite over time, revealing dynamic vs. stable synapses (Pagès et al., 2015).

Koskinen and Hotulainen evaluate three methods that can retrieve information on actin dynamics, to obtain an in-depth understanding of synapse functionality and plasticity. The principles of these three methods —Fluorescent Recovery After Photobleaching, PhotoActivable Green Fluorescent Protein fluorescence decay and fluorescence anisotropy— are explained, with their respective analysis methods, advantages and limitations. Furthermore, they propose using fluorescent anisotropy for actin bundling analysis (Koskinen and Hotulainen, 2014).

Finally, a philosophical perspective article by Malanowski and Craver explores the topic of spine function. Their argument is based on the idea, developed recently by philosophers using examples from neurobiology and molecular biology, that mechanisms provide a fruitful framework for causal explanation. Explanation in such a framework can be etiological, constitutive or contextual, and allows a comprehensive bridge to be built between levels (e.g., molecule, neuron, network, behavior, cognition). This article centers on the conditions and methods by which we could attribute a function or role to spines. The article, which shows that spines may play a role at many levels of organization and in many distinct causal systems, illustrates how philosophy can provide a relevant analysis of biological problems and rationalize concepts which may help neurobiologists to advance in their field of research (Malanowski and Craver, 2014).

In summary, this volume brings together a series of outstanding articles, dealing with some of the most recent ideas concerning the structure and function of dendritic spines. We hope this collection provides the reader with valuable information regarding this area of research and promotes further understanding of these fascinating structures which enable brain function.

## Funding

R.B.-P. was supported by the Ministerio de Economia y Competitividad (CSIC).

### Conflict of interest statement

The authors declare that the research was conducted in the absence of any commercial or financial relationships that could be construed as a potential conflict of interest.

## References

[B1] Adrian M. Kusters R. Wierenga C. J. Storm C. Hoogenraad C. C. Kapitein L. C. (2014). Barriers in the brain: resolving dendritic spine morphology and compartmentalization. Front. Neuroanat. 8:142. 10.3389/fnana.2014.00142 25538570 PMC4255500

[B2] Araya R. (2014). Input transformation by dendritic spines of pyramidal neurons. Front. Neuroanat. 8:141. 10.3389/fnana.2014.00141 25520626 PMC4251451

[B3] Bernardinelli Y. Nikonenko I. Muller D. (2014). Structural plasticity: mechanisms and contribution to developmental psychiatric disorders. Front. Neuroanat. 8:123. 10.3389/fnana.2014.00123 25404897 PMC4217507

[B4] Chen C.-C. Lu J. Zuo Y. (2014). Spatiotemporal dynamics of dendritic spines in the living brain. Front. Neuroanat. 8:28. 10.3389/fnana.2014.00028 24847214 PMC4023020

[B5] Cheng C. Trzcinski O. Doering L. C. (2014). Fluorescent labeling of dendritic spines in cell cultures with the carbocyanine dye “DiI.” Front. Neuroanat. 8:30. 10.3389/fnana.2014.00030 24847216 PMC4023042

[B6] DeFelipe J. (2015). The dendritic spine story: an intriguing process of discovery. Front. Neuroanat. 9:14. 10.3389/fnana.2015.00014 25798090 PMC4350409

[B7] Dotti C. G. Esteban J. A. Ledesma M. D. (2014). Lipid dynamics at dendritic spines. Front. Neuroanat. 8:76. 10.3389/fnana.2014.00076 25152717 PMC4126552

[B8] Elston G. N. Fujita I. (2014). Pyramidal cell development: postnatal spinogenesis, dendritic growth, axon growth, and electrophysiology. Front. Neuroanat. 8:78. 10.3389/fnana.2014.00078 25161611 PMC4130200

[B9] Frotscher M. Studer D. Graber W. Chai X. Nestel S. Zhao S. (2014). Fine structure of synapses on dendritic spines. Front. Neuroanat. 8:94. 10.3389/fnana.2014.00094 25249945 PMC4158982

[B10] Kelly E. A. Opanashuk L. A. Majewska A. K. (2014). The effects of postnatal exposure to low-dose bisphenol-A on activity-dependent plasticity in the mouse sensory cortex. Front. Neuroanat. 8:117. 10.3389/fnana.2014.00117 25374513 PMC4205826

[B11] Koskinen M. Hotulainen P. (2014). Measuring F-actin properties in dendritic spines. Front. Neuroanat. 8:74. 10.3389/fnana.2014.00074 25140131 PMC4122166

[B12] Levy A. D. Omar M. H. Koleske A. J. (2014). Extracellular matrix control of dendritic spine and synapse structure and plasticity in adulthood. Front. Neuroanat. 8:116. 10.3389/fnana.2014.00116 25368556 PMC4202714

[B13] Lüscher C. Kiss J. Z. Holtmaat A. (2015). Dominique Muller (1956-2015). Neuron 87,12–13. 10.1016/j.neuron.2015.06.027 26349101

[B14] Malanowski S. Craver C. F. (2014). The spine problem: finding a function for dendritic spines. Front. Neuroanat. 8:95. 10.3389/fnana.2014.00095 25309340 PMC4159972

[B15] Mancuso J. J. Cheng J. Yin Z. Gilliam J. C. Xia X. Li X.. (2014). Integration of multiscale dendritic spine structure and function data into systems biology models. Front. Neuroanat. 8:130. 10.3389/fnana.2014.00130 25429262 PMC4228840

[B16] Pagès S. Cane M. Randall J. Capello L. Holtmaat A. (2015). Single cell electroporation for longitudinal imaging of synaptic structure and function in the adult mouse neocortex *in vivo*. Front. Neuroanat. 9:36. 10.3389/fnana.2015.00036 25904849 PMC4387926

[B17] Segal M. Korkotian E. (2014). Endoplasmic reticulum calcium stores in dendritic spines. Front. Neuroanat. 8:64. 10.3389/fnana.2014.00064 25071469 PMC4089118

[B18] Spiga S. Mulas G. Piras F. Diana M. (2014). The “addicted” spine. Front. Neuroanat. 8:110. 10.3389/fnana.2014.00110 25324733 PMC4183114

[B19] Stawarski M. Stefaniuk M. Wlodarczyk J. (2014). Matrix metalloproteinase-9 involvement in the structural plasticity of dendritic spines. Front. Neuroanat. 8:68. 10.3389/fnana.2014.00068 25071472 PMC4091410

[B20] Takasaki K. Sabatini B. L. (2014). Super-resolution 2-photon microscopy reveals that the morphology of each dendritic spine correlates with diffusive but not synaptic properties. Front. Neuroanat. 8:29. 10.3389/fnana.2014.00029 24847215 PMC4019874

[B21] Xu X. Miller E. C. Pozzo-Miller L. (2014). Dendritic spine dysgenesis in Rett syndrome. Front. Neuroanat. 8:97. 10.3389/fnana.2014.00097 25309341 PMC4159975

